# Induced Pluripotent Stem Cells in Dental and Nondental Tissue Regeneration: A Review of an Unexploited Potential

**DOI:** 10.1155/2020/1941629

**Published:** 2020-03-29

**Authors:** Israa Ahmed Radwan, Dina Rady, Marwa M. S. Abbass, Sara El Moshy, Nermeen AbuBakr, Christof E. Dörfer, Karim M. Fawzy El-Sayed

**Affiliations:** ^1^Oral Biology Department, Faculty of Dentistry, Cairo University, Cairo, Egypt; ^2^Stem Cells and Tissue Engineering Research Group, Faculty of Dentistry, Cairo University, Cairo, Egypt; ^3^Clinic for Conservative Dentistry and Periodontology, School of Dental Medicine, Christian Albrechts University, Kiel, Germany; ^4^Oral Medicine and Periodontology Department, Faculty of Dentistry, Cairo University, Cairo, Egypt

## Abstract

Cell-based therapies currently represent the state of art for tissue regenerative treatment approaches for various diseases and disorders. Induced pluripotent stem cells (iPSCs), reprogrammed from adult somatic cells, using vectors carrying definite transcription factors, have manifested a breakthrough in regenerative medicine, relying on their pluripotent nature and ease of generation in large amounts from various dental and nondental tissues. In addition to their potential applications in regenerative medicine and dentistry, iPSCs can also be used in disease modeling and drug testing for personalized medicine. The current review discusses various techniques for the production of iPSC-derived osteogenic and odontogenic progenitors, the therapeutic applications of iPSCs, and their regenerative potential in vivo and in vitro. Through the present review, we aim to explore the potential applications of iPSCs in dental and nondental tissue regeneration and to highlight different protocols used for the generation of different tissues and cell lines from iPSCs.

## 1. Introduction

Embryonic stem (ES) cells are pluripotent cells derived from the inner cell mass of the blastocyst. They can give rise to tissues derived from the three germ layers and are regarded as a renewable potent cell source for the regeneration of all body tissues [1–4]. However, ES usage in regenerative medicine faces a lot of obstacles as their isolation requires destruction of human embryos which raises justified ethical objections. ES can also elicit an immune response upon transplantation in patients [5]. In 2006, Takahashi et al. [6] demonstrated that mature differentiated cells can be reprogrammed and dedifferentiated into embryonic-like cells, with ES-like properties. Mature murine fibroblast cell lines were reversed into pluripotency via retroviral transduction of 4 transcription factors, POU domain class 5 transcription factor 1 (Oct3/4), the sex-determining region Y-box2 (Sox2), Kruppel-like factor 4 (Klf4), and myelocytomatosis oncogene (c-Myc), giving rise to induced pluripotent stem cells (iPSCs). Those four transcription factors (also referred to as OSKM factors) were postulated to be responsible for the maintenance of ES inherent pluripotency. Over the subsequent years, iPSCs were generated from a variety of adult tissues [7–9] and were similar to ES in morphology, proliferative rates, surface antigens, expressed genes, and in vivo teratoma formation [6].

## 2. iPSC Source and Generation (Reprogramming) Methods

**Figure 1:**
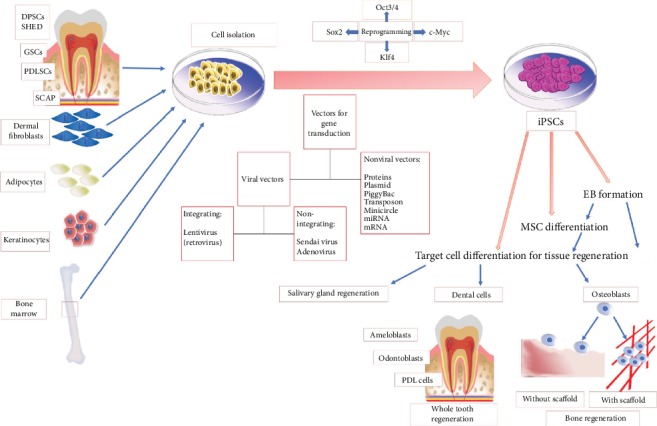
Diagram summarizing iPSC source, methods of gene transduction, and iPSC differentiation. Dental pulp stem cells (DPSCs), stem cells from exfoliated deciduous teeth (SHED), gingival stem cells (GSCs), stem cells from apical dental papilla (SCAP), embryoid bodies (EB), mesenchymal stem cells (MSCs), and induced pluripotent stem cells (iPSCs).

iPSCs were successfully generated from different dental and nondental tissues (Figure 1) including fibroblasts, keratinocytes, melanocyte blood cells, bone marrow cells, adipose cells, tissue-resident progenitor cells, and gingival and periodontal ligament fibroblasts [10–13] via transduction of Oct3/4, Sox2, and Klf4 [14, 15]. iPSCs were also successfully generated from dental pulp stem cells (DPSCs) [16–18], stem cells from human exfoliated deciduous teeth (SHED) [18, 19], and stem cells from apical dental papilla [18]. Gingival fibroblast-derived iPSCs were considered to be advantageous over dermal fibroblasts (DF) as they could be easily acquired during routine dental treatment and were effectively reprogrammed into iPSCs [14].

As mentioned above, generation of iPSCs depends on the transduction of specific transcription factors into the somatic cell genome via vectors for its reprogramming [20]. Vectors used during the generation of iPSCs can be divided into integrative viral vectors, integrative free vectors, and nonviral vectors [21]. Originally, lentivirus (a retrovirus), an integrating viral vector, was used for iPSC generation with high reprogramming efficacy [6]. Despite offering a high transduction ability, integrating viral vectors insert their whole genome into recipient cells and may introduce oncogenes or genetic mutations into the host cells [22] (Figure 1).

Nonintegrating viruses, such as Sendai virus and adenovirus, were subsequently introduced in an attempt to overcome these drawbacks [23]. Tashiro et al. [24] compared four types of promoters (RSV, CMV, cytomegalovirus enhancer/b-actin (CA), and elongation factor-1a (EF-1a)) using adenovirus vectors for iPSC induction. An adenovirus vector containing EF-1a and CA promoter efficiently transduced transgenes into mouse iPSCs, without a decrease in pluripotency or viability. An optimized adenovirus vector that was developed by the authors enhanced adipocyte and osteoblast differentiation, confirmed by significant gene expressions of peroxisome proliferator-activated receptor c and runt-related transcription factor 2 (RUNX2), respectively, by iPSCs.

To avoid an increased risk of tumor generation and chromosomal instability, nonviral vectors were subsequently introduced for the somatic reprogramming process, including proteins, plasmid, piggyBac transposon, minicircle vector, miRNA, and mRNA [25–30]. Gene-editing technologies like CRISPR/Cas9, zinc finger nucleases, and transcription activator-like effector nucleases (TALENs) were additionally employed for genome editing of iPSCs to introduce certain traits for disease modeling and cancer research or to alter their gene expression for possible application in the field of regenerative medicine [31].

## 3. Assessment of Pluripotency

Following iPSC generation, cells have to be assessed via pluripotency assays, including morphological and histological analysis, and certain gene expressions, proving their ability to differentiate into tissues derived from the three germ layers and teratoma formation [32]. Teratoma assays involve injection of iPSCs into immunocompromised experimental animals and subsequent formed tissue analysis to assure teratoma formation [33]. Alternatively, in vitro embryoid body (EB) generation can be used to ascertain pluripotency; EB is a mass of cells derived from all three germ layers [32], generated from iPSCs upon culturing in proper media [32, 34, 35]. EB generation encompasses the homogeneous method as the liquid suspension method and the heterogeneous method as the hanging drop culture. While the heterogeneous method is considered the easiest way to generate EB, the resulting cell masses are largely heterogeneous in size [36], which are irreproducible [37] and negatively affect subsequent iPSC differentiation towards a specific cell line [38]. The homogeneous method, on the other hand, creates cell masses of more homogeneous, uniform sizes that subsequently enhance cell viability and facilitate their subsequent differentiation into specific cell lines [33, 39]. To avoid tumor formation, prior to implantation, iPSCs are either differentiated into mesenchymal stem cells (MSCs) or targeted tissue cell types with or without EB formation (Figure 1).

## 4. iPSCs in Dental and Nondental Tissue Regeneration (Table 1)

### 4.1. iPSCs and Bone Regeneration

Although autogenous bone graft remains to be the gold standard for reconstruction of bone defects [40], it carries the risk of bone resorption and donor site infection and the graft may not always be available in sufficient amounts [41]. iPSC technologies may provide a suitable alternative to autogenous grafting, whereby patients' somatic cells are induced into bone-forming cells that are loaded on an appropriate scaffold in combination with proper bioactive molecules for bone tissue engineering [42]. To induce osteogenic differentiation of iPSCs, a variety of agents were proposed in isolation or combination, including osteogenic media, ascorbic acid, b-glycerophosphate, dexamethasone, bone morphogenetic proteins (BMPs), and vitamin D_3_ [43–46]. Osteogenic differentiation is followed by proper characterization of generated bone cells through their expression of osteogenesis-related genes (RUNX2, osteopontin (OPN), osterix (OSX), osteocalcin (OCN), and collagen type I (COL1A1)) [47–50] in addition to the evaluation of in vitro mineralization and alkaline phosphatase (ALP) activity [51, 52].

Osteogenic potential of human iPSCs was demonstrated on polymeric nanofibrous polyethersulfone (PES) scaffold with upregulated expressions of osteogenic genes and alkaline phosphatase activity in vitro [48, 53]. The expression of key osteoblast-related genes in undifferentiated iPSCs was nearly 30 times higher than in undifferentiated ES cells. On the contrary, the expression of the same genes in ES- and iPSC-derived osteoblasts was not significantly different except for OPN and COL1A1, which were significantly higher in iPSC-derived osteoblasts [51]. Evidence revealed that ES cells and iPSCs generated from transgenic mice expressing rat 2.3 kb type I collagen promoter-driven green fluorescent protein (Col2.3GFP) successfully differentiated into osteoblast lineage cells that expressed Col2.3GFP in vitro [54]. Gene expression profiles proved that ES- and iPSC-derived osteoblasts resemble osteoblasts present in the calvaria [54].

The osteoinductive properties of iPSC-derived bone cells and their capability in treating bone defects were further assessed in vivo by their implantation into a severe combined immunodeficiency (SCID) mouse model. Bone formation was confirmed four weeks following implantation by soft X-ray images [43], X-ray microcomputed tomography (*μ*CT) [55], cone beam computed tomography imaging [49], and histological tissue specimens [43, 47–52]. In a cleidocranial dysostosis model, the mutation in RUNX2 gene was repaired in iPSCs derived from mucosal tissues of affected patients. The reverted cells revealed marked upregulation of osteoblast differentiation markers after being cultured in OM for nine days. Loading the differentiated osteoblasts originating from iPSCs with a corrected mutation on a peptide nanofiber scaffold and implanting them into SCID rats' calvarial bone defects revealed reossification four weeks after transplantation with a significant increase in bone volume and bone mineral content [52]. Similarly, osteogenic cells differentiating from EB derived from iPSCs showed positive results in bone regeneration and healing following implantation in the rats' critical-sized calvarial defect [53, 56, 57] and long bone segmental defect rat model [57] after being loaded on polymeric nanofibrous PES scaffold [53], fibrin glue scaffold [57], hydroxyapatite (HA)/b-tricalcium phosphate scaffold [57], or self-assembling peptide nanofiber hydrogel scaffold [56]. Moreover, iPSCs differentiated into functional osteoblasts and demonstrated a bone regenerative effect comparable to human bone marrow- (BM-) MSCs in vivo [57].

#### 4.1.1. Osteogenic Potential of iPSCs-MSCs Obtained through EB Formation

This method entails the differentiation of MSCs from EB-derived iPSCs. It was suggested to possess notable advantages over direct differentiation of iPSCs into osteoblasts, with the resulting osteogenic cells demonstrating a significant upregulation of osteoblast-related genes including ALP, RUNX2, COL1A1, and OCN [58, 59]. Several factors were demonstrated to influence the osteogenic potential of iPSC-derived MSCs including the incorporation of retinoic acid, transforming growth factor-beta (TGF-*β*) [60, 61], or metformin into the culture media [62] as well as coseeding with other cell types [63–65]. The suspension time of EB and genetic modification of iPSCs-MSCs also proved to affect their osteogenic capability [66–68]. Culturing EB generated from dermal fibroblast iPSCs in media supplemented with TGF-*β* induced MSC differentiation. Two populations of MSCs were recognized, early MSCs that migrated from EB during days 2–5 and late MSCs that crawled from EB during days 5–8. The two iPSC-derived MSC populations and BM-MSCs were transduced with BMP-6 plasmid. Resulting cells were either suspended in fibrin gel and injected into thigh muscles of SCID rats or loaded on collagen scaffolds and implanted in a nonunion radial fracture SCID rat model. No or limited bone formation was acquired upon ectopic injection of BMP-6-late MSCs, while opposite results were obtained upon injecting BMP-6-early MSCs. It was concluded that iPSCs-MSCs obtained at early EB suspension time possessed a more pronounced stem cell phenotype and were capable of ectopic bone formation, whereas those cells obtained later acquired a more differentiated phenotype of osteoblasts and were capable of significant bone formation in vivo [68].

Similarly, genetic modification of human iPSCs-MSCs by either BMP-2 or NELL1 overexpression, followed by seeding of the modified cells on calcium phosphate cement (CPC) scaffold immobilized with RGD (Arg-Gly-Asp), showed significantly high expression of RUNX2, OCN, and COL1A1 [66, 67]. Additionally, human iPSCs-MSCs that were either osteoinduced or transduced with BMP-2 demonstrated high expression levels of osteoblast-related genes [69]. Incorporating retinoic acid combined with TGF-*β*1 or TGF-*β*1 into murine iPSC-derived EB culture media enhanced mineralization and osteogenic differentiation [60, 61]. Additionally, human iPSCs-MSCs cultured in the presence of metformin and seeded on CPC scaffolds showed upregulated expression of osteoblast-related genes and proteins as well as increased mineralization. Induction of adenosine monophosphate- (AMP-) activated protein kinase phosphorylation concomitant with increased RUNX2 expression was also evident [62]. Moreover, coseeding of human iPSCs-MSCs with human umbilical vein endothelial cells (HUVECs) on CPC scaffolds [63, 64] or coseeding with pericytes [65] enhanced osteogenesis and vascularization in vitro and in vivo with an upregulation expression of osteogenic (ALP, OCN, and COL1A1) and angiogenic genes (vascular endothelial growth factor (VEGF) and vascular endothelial cadherin).

Antibody-mediated osseous regeneration was recently described to impact in vivo bone regeneration. Human iPSCs-MSCs were combined with 3G7, an anti-BMP-2 antibody, that were hypothesized to facilitate the engagement of BMP-2 to their receptors on iPSCs-MSCs. 3G7 and iPSCs-MSCs were subsequently loaded on biocompatible, biodegradable alginate microbeads that were injected subcutaneously in rats. In vivo enhanced bone formation, mineralization, and vascularization associated with in vitro enhanced osteogenic differentiation were mediated through activation of the BMP-2/Smad1/RUNX2 pathway [70].

Biofunctionalization of the scaffold was further suggested to promote human iPSCs-MSCs osteogenic differentiation and vascularization, where human iPSCs-MSCs seeded on CPC scaffolds, treated with biofunctional agents and bioactive peptides [71–73] as well as murine iPSCs-MSCs seeded on biomimetic nanofibers of hydroxyapatite/collagen/chitosan (HA/COL/CTS), showed upregulation of RUNX2, OSX, ALP, and COL1A1 gene expression levels [74]. Furthermore, outgrowing cells from mouse iPSCs cultured on different polystyrene substrate topographies displayed upregulation of COL1A1 and RUNX2 [75]. Human iPSCs-MSCs seeded on microporous CPC scaffolds using polyethylene glycol (PEG) particles showed upregulation of RUNX2, COL1A1, ALP, OPN, and platelet-derived growth factor receptor-beta (PDGF-R-*β*) [76]. Similarly, human iPSCs-MSCs seeded on CPC [62, 77–79] or poly lactic-co-glycolic acid/poly L-lactic acid (PLGA/PLLA) scaffold combined with macrophages [80] or fast degradable alginate microbeads [69] showed high expression of osteoblast-related genes. Moreover, murine iPSC-derived MSCs seeded onto three-dimensional gelatin scaffold revealed upregulation of several osteoblast-related genes in vitro and in vivo, following subcutaneous implantation in rats [81]. Demonstrating the key role of osteoprotegerin/receptor activator of nuclear factor *κ* B ligand (OPG/RANKL) in orchestrating osteoblastic and osteoclastic action in bone remodeling, human iPSCs-MSCs were cocultured with iPSCs-macrophages committed to osteoblastogenesis and osteoclastogenesis, respectively, on HA-based PLGA/PLLA 3D scaffolds. Enhanced expression of bone-related genes upon monoculturing human iPSCs-MSCs on HA-5 PLGA/PLLA was demonstrated as compared to HA-0 PLGA/PLLA. Coculturing induced upregulated expression of late osteogenic markers (OPN and OCN) and downregulated expression of early osteogenic markers (COL1A1, ALP, and RUNX2). Similar results were attained in vivo through implantation of HA-PLGA/PLLA scaffold loaded with human iPSCs-MSCs and iPSCs-macrophages subcutaneously in rodents [80].

#### 4.1.2. Osteogenic Potential of iPSCs-MSCs Obtained without EB Formation

Another method proposed to obtain iPSCs-MSCs relies on the dissociation of iPSC colonies, without prior formation of EB, into a single cell suspension. The resulting cells are characterized as MSCs, either through flow cytometry or through cell passaging protocols, followed by osteogenic differentiation [82–84]. Dimethyloxaloylglycine (DMOG) promoted iPSCs-MSCs derived from human foreskin fibroblast angiogenesis in critical-sized calvarial rat defects [85]. DMOG enhanced the expression of angiogenic factors (hypoxia-inducible factor 1-*α* (HIF-1*α*) and VEGF) through PI3K/Akt intracellular pathway activation, with improved bone formation.

The osteogenic potential of iPSCs-MSCs in combination with different scaffolds was investigated in several studies [55, 86–90]. The subcutaneous implantation of osteoinduced episomal-iPSCs (generated using an episomal vector) derived from BM stromal cells and retro-iPSCs (generated using a retroviral vector) derived from DF cultured on decellularized bone scaffold in SCID mice for 12 weeks revealed high mineral content in the episomal-iPSCs as compared to retro-iPSCs [86]. On the other hand, retro-iPSCs displayed the formation of a uniform bone-like matrix with embedded cells, while episomal-iPSCs exhibited areas of dystrophic calcification [86]. The osteogenic potential of human fibroblast-derived iPSCs was evaluated in vitro and in vivo on synthetic polymer polycaprolactone (PCL) scaffold or PCL scaffold functionalized with natural polymer hyaluronan and ceramic tricalcium phosphate ceramic poly (3-hexylthiophene (TCP-PHT)) [90]. The osteoinduced iPSCs revealed a significant increase in ALP activity and calcium deposition on PHT scaffold in vitro as well as ectopic bone formation in vivo in comparison to PCL. Moreover, human fibroblast-derived iPSCs on PCL nanofibers alone or combined with nano-HA showed an increased expression of osteogenic genes (RUNX2, ALP, COL1A1, and OCN) in both scaffolds, even though they were expressed at a different time intervals, OCN was highly expressed in PCL-nano-HA in comparison to PCL scaffolds [89]. Similarly, the incorporation of short hydrophilic peptide H1 derived from connective tissue growth factor in a core silk fibroin (SF) combined with HA derived from poly (L-lactic acid-co-*ε*-caprolactone) (PLCL) resulted in increased proliferation and osteogenic differentiation of iPSCs-MSCs derived from human fibroblasts [55].

The interaction between HA/TCP ceramic particles and iPSCs-MSCs was subsequently investigated in vivo [87, 88]. Rhesus macaques' iPSC-derived mesodermal stromal-like cells mixed with HA/TCP demonstrated robust bone formation when implanted subcutaneously for eight weeks [87]. Furthermore, the osteogenic potential of iPSCs-MSCs from gingival fibroblasts, periodontal ligament cells, and human lung combined with HA/TCP was compared following implantation in SCID mice subcutaneously [88]. Although the three types of iPSCs-MSCs were able to form mineralized tissue, iPSCs-MSCs derived from periodontal ligament cells showed superior capability to form mature bone and connective tissue, which led to a controversial assumption that even after induction, iPSCs may retain epigenetic memory of their origin [91]. The combination of HA derived from PLCL with osteoinductive peptide H1 in a core SF and iPSCs-MSCs derived from human fibroblasts resulted in faster bone formation in vivo as compared to SF/PLCL following eight weeks of implantation in calvarial mouse defects [55].

Yet, although most of the aforementioned studies highlighted the osteogenic potential of iPSCs-MSCs in bone regeneration, Chijimatsu et al. reported that MSCs derived from iPSCs-neural crest cells failed to repair rat osteochondral knee defects in vivo despite their demonstrated chondrogenic and osteogenic capacity comparable to human BM-MSCs in vitro [92].

#### 4.1.3. Osteogenic Differentiation Capability of iPSCs Compared to Other Types of Cells

The osteogenic differentiation ability of iPSCs-MSCs in comparison to MSCs was examined in a variety of studies [86, 93–95]. A study on iPSCs showed a delayed expression of osteogenic markers such as COL1A1 and bone sialoprotein (BSP) as well as weaker osteoblastic differentiation and mineral deposition, compared to human BM-MSCs in vitro [57]. Human fibroblast-derived iPSCs reprogrammed by mRNA (mRNA-iPSCs) or polycistronic lentiviral vector (lenti-iPSCs) were compared to BM-MSCs [95]. Both methods of transduction produced cells that were similar in their morphology and surface antigen to BM-MSCs. lenti-iPSCs revealed faster and more homogeneous calcium staining than mRNA-iPSCs. Although the expression of RUNX2, ALP, and OCN was stronger in BM-MSCs as compared to iPSCs-MSCs, the opposite was demonstrated for COL1A1 expression. Both iPSCs-MSCs showed osteogenic efficacy comparable to BM-MSCs. Similarly, osteoinduced mouse iPSCs-MSCs revealed the same surface antigen profile and higher osteogenic differentiation as BM-MSCs [93]. ALP, OSX, RUNX2, and OCN were intensely upregulated in osteoinduced iPSCs-MSCs aside from the formation of a mineralized matrix at day 14 of osteogenic induction. retro-iPSCs and episomal-iPSCs exhibited higher ALP gene expression than human ES cells [86]. Moreover, the osteogenic potential of iPSCs-MSCs derived from either human deciduous teeth or human DF was higher than that of osteoinduced SHED [94]. iPSCs-MSCs derived from equine fibroblast iPSCs were compared to MSCs derived from newborn foals' umbilical cord blood (CB-MSCs) [96]. Von Kossa and alizarin red staining of iPSCs-MSCs showed early mineralization indicating early osteogenesis which was consistent with the results obtained from CB-MSCs.

Similarly, Ardeshirylajimi et al. [97] compared the biological behavior and osteogenic differentiation potential of human iPSCs and adipose tissue (AT-MSCs). iPSCs confirmed high osteogenic differentiation potential and superior ALP activity and mineralization level. Notably, AT-MSCs expressed greater levels of RUNX2, while iPSCs expressed higher levels of OCN and osteonectin during differentiation which may be a result of their increased proliferation rate compared to AT-MSCs [97]. In vivo comparison of osteogenic potentials between adipose-derived stem cells (ASCs) and ASC-iPSCs loaded on nano-HA gelatin cryogel scaffolds revealed a superior osteogenic differentiation with enhanced osteogenic marker expression of COL1A1 and RUNX2 in the ASC-iPSCs group, proposing ASC-iPSCs as an alternative cell source in bone tissue engineering with a good differentiation ability [98].

On the other hand, the osteogenic potential of iPSCs derived from human skin fibroblasts was compared to iPSCs derived from BM-MSCs cultured on HA/TCP implanted subcutaneously in nude mice [99]. No differences in bone formation were revealed between iPSCs from different origins. In addition, the bone regeneration ability of adipose-derived stromal cells- (AS-) iPSCs was compared to human ES cells cultured on HA-coated PLGA scaffold with or without releasing BMP-2 in calvarial mouse defects [100]. Greater bone regeneration as well as upregulation of osteogenic markers was found in both AS-iPSCs and ES cells loaded on HA-PLGA releasing BMP-2 as compared to nonreleasing BMP-2 [100].

#### 4.1.4. Factors to Improve the Osteogenic Potential of iPSCs (Figure 2)

Exploring the therapeutic potential of iPSCs-MSCs in dental and nondental tissue regeneration entails the optimization of the factors that would enhance their osteogenic potential for future clinical applications. Genes, isozymes, laser application, suspension time of EBs, transduction method, natural antioxidant and anticancer products, and constituents of the scaffold material are factors that could enhance or affect the osteogenic potential of iPSCs. In order to attain iPSC osteogenic commitment, various inductive factors were applied including chemical inducers, biomolecules [101–103], growth factors [100], gene modification [104], two-dimensional culture environment [105], and modified three-dimensional scaffolds [100, 101, 106–108]. Tissue-nonspecific alkaline phosphatase (TNAP) was demonstrated to influence the osteogenic differentiation potential of iPSCs, where TNAP-positive cells isolated from human EBs derived from iPSCs and cultured in osteogenic media expressed high levels of OSX, RUNX2, COL1A1, BSP, and OCN as well as generated mineralized nodules and revealed a significant expression of osteocyte marker genes, including sclerostin, neuropeptide Y, and reelin [109]. Similarly, extremely low-frequency electromagnetic field (ELF-EMF) (50 Hz and 1.5 mT) also significantly improved the osteogenic potential of iPSCs [110]. Resveratrol a natural polyphenol found largely in red grapes, nuts, pomegranates, and red wine [111] was also found to facilitate osteogenic differentiation of iPSCs, with increased osteogenic gene expression and mineralization content [112]. Growth factors such as recombinant human- (rh-) BMP-2 have been shown to positively modulate osteogenic transformation of iPSCs. Adding rh-BMP-2 to the osteogenic media improved the osteogenic potential of iPSCs derived from human AS through significant upregulation of osteogenic markers RUNX2 and OCN [100]. In vitro results showed that 3 wt/vol% nano-HA in chitosan/gelatin (CG) and miRNAs increased the expression of osteogenic-related genes [49, 50], formed bone-like tissue in vivo [49], and upregulated the OCN and OPN protein expression on day 21 after culturing [50].

Even though growth factors can endorse the osteogenic differentiation of iPSCs, their effects are limited due to their short half-lives and uncontrolled degradation. In contrast, gene modification of iPSC-derived cells can attain a long-term effect via retaining a relatively stable local concentration of these factors [113]. Certain genes such as nuclear matrix protein SATB2 have been transduced into iPSCs to promote osteodifferentiation [104]. An efficacious strategy for differentiating human iPSCs into osteoblasts involves using four small molecules including CHIR99021 (CHIR), cyclopamine (Cyc), smoothened agonist (SAG), and helioxanthin-derivative 4-(4-methoxyphenyl) pyrido [4′,3′:4,5] thieno [2,3-b] pyridine-2-carboxamide (TH) under chemically well-defined conditions [114]. Ex vivo gene therapy of SATB2-modified iPSCs increased the levels of calcium nodule formation, ALP activity, and osteogenic genes in vitro. Subsequent implantation of the transduced cells on silk scaffold encouraged bone regeneration in critical-sized calvarial defects [104]. On the contrary, iPSCs derived from tail-tip fibroblasts of Alox5 knockout mouse demonstrated significant downregulation of early and late osteogenic gene levels with significant upregulation of adipogenic markers. Still, loading Alox5-KO-iPSCs on collagen/chitosan/hydroxyapatite scaffolds induced significantly less new bone formation in rat cranial critical-sized defects as compared to wild-iPSCs [115].

Interestingly, iPSC origin demonstrated no effect on iPSC osteogenic potential. The osteogenic differentiation properties of human iPSCs derived from BM-MSCs and DFs demonstrated no marked differences in gene expression profiles as well as in the methylation profile. Moreover, the chondrogenic and osteogenic differentiation properties of iPSCs from different cells' origin showed no significant differences, although a higher tendency was reported in DF-derived iPSCs [91]. Yet, different reprogramming methods could affect the osteogenic differentiation of iPSCs [86]. iPSCs derived from DF reprogrammed by retroviral vectors (retro-iPSCs) or Sendai virus (Sendai-iPSCs) cultured on decellularized bone scaffold in perfusion bioreactors demonstrated a new bone-like matrix with the highest cell density in Sendai-iPSCs, while retro-iPSCs showed poor osteogenic differentiation [86].

Human iPSCs derived from human embryonic kidney-EB were utilized to compare the osteoinductive properties of 3D nanofibrous scaffold of polyvinylidene fluoride (PVDF) with 2D scaffold [116] as well as to assess electrospun poly (3-hydroxybutyrate-co-3-hydroxyvalerate) (PHBV) nanofiber scaffold [117]. iPSCs revealed significantly high ALP activity, calcium content, and osteogenic-related genes after seeding on 3D PVDF [116] and PHBV scaffolds [117]. Moreover, OCN and OPN protein expressions were elevated on day 21 after cell seeding [116, 117]. Utilizing different ratios from nano-HA [49] or different miRNAs (miR-22 and miR-126) [50] in chitosan/gelatin (CG) scaffold or electrospun PCL nanofiber scaffold, respectively, was also reported to affect the osteogenic differentiation of human iPSCs. Furthermore, incorporating basic fibroblast growth factor (bFGF) in PCL-PVDF scaffold [47] or polyphosphate (poly-P) in PCL/PLLA electrospun scaffolds [118] or graphene oxide (GO) in PVDF nanofibers [119] or platelet-rich plasma in PVDF/collagen nanofibrous scaffolds [120] significantly increased the survival rate of iPSCs and upregulated ALP activity, mineralization content, and expression of preosteoblast- and osteoblast-related genes in iPSCs loaded on PCL-PVDF (bFGF), PCL-PLLA (poly-P), PVDF-GO, or PVDF/Col/PRP scaffolds [47, 118–120]. A xeno-free nanofiber scaffold conjugated with vitronectin peptide upheld pluripotency and proliferation of seeded human iPSCs. Interestingly, this osteogenic culture system promoted direct osteodifferentiation of human iPSCs, as confirmed by the cellular morphology, ALP assay, and RT-PCR analysis combined with immunofluorescence results [101]. A recent report confirmed the osteogenic differentiation of human iPSCs into osteoblast-like cells with enhanced calcified nodule formation under the influence of retinoic acid in vitro and membranous bone tissue formation in vivo without scaffolds [103]. Under osteogenic conditions, human iPSCs cultured on PCL scaffolds confirmed osteogenesis by OPN detection using quantitative PCR and by western blotting. Further subcutaneous implantation in mice revealed marked calcium deposition and positive OCN immunostaining, with no signs of teratoma formation, following the osteogenic induction of human iPSCs [106]. The osteogenic potential of human iPSC-derived mesodermal progenitor cells (hiPSC-MP) on decellularized tissue matrices as scaffolding materials and human bone scaffolds in osteogenic medium under dynamic conditions was compared in perfusion bioreactors. Both scaffolds equally promoted cell viability and mineralized tissue formation [108]. Peptide-decorated 2D culture microenvironment developed through polydopamine (pDA) chemistry with subsequent carboxymethyl chitosan successfully promoted osteogenic differentiation of human iPSCs in vitro [105]. These results were supported by enhanced ALP activity, gene expression, and corresponding protein expression as well as the amount of calcium deposition [105]. Human iPSCs isolated from clinically discarded human gingival tissues were seeded on both sphere-shaped or rod-shaped nano-HA/CG scaffolds. Notably, the sphere-shaped nano-HA in HA/CG scaffolds greatly improved the osteogenic differentiation of human iPSCs as compared to rod-shaped. Consequently, human iPSCs and sphere-shaped nano-HA/CG composites generated a significant amount of bone in vivo [121].

Adenosine-induced differentiation of human iPSCs (Ad-iPSCs) loaded on poly (ethylene glycol) diacrylate-co-acryloyl 6-aminocaproic acid (PEGDA-co-A6ACA) macroporous hydrogel into functioning osteoblast, in growth medium lacking any other osteoinductive factors, revealed progressive dense bone tissue formation. Furthermore, Ad-iPSCs implanted in critical-sized cranial bone defects in mice showed uniform hard tissue formation all over the cranial defect that was integrated with the adjacent bone without teratoma formation [102]. Moreover, ex vivo two-dimensional and three-dimensional cultures and mineralized gelatin methacrylate- (GelMA-) based matrices containing CaP mineral endorse the osteogenic differentiation of human iPSCs in osteoinductive factors free growth medium via the dissociation of Ca^2+^ and PO_4_^3-^ ions in a permissive environment through various signaling pathways [107]. Similarly, ectopically implanted human iPSCs seeded on coral scaffolds in mice demonstrated the expression of bone-like structures through the release of osteoinductive factors including BMPs [122]. Paradoxically, the rapid disappearance of human iPSCs due to early cell death was associated with an increase in the osteogenic genes. To settle these conflicting trends, the authors investigated the paracrine effect of bioactive CM from human iPSCs. Interestingly, human iPSC CM promoted the osteogenic differentiation of human MSC osteogenic differentiation as well as upregulated the expression of BMP-2, BMP-4, and BMP-6 genes and enhanced extracellular matrix mineralization [122].

### 4.2. iPSCs and Salivary Gland Regeneration

iPSC therapeutic and regenerative potentials were exploited in the treatment of salivary glands' diseases. In an in vivo study, iPSCs were utilized for treating salivary gland carcinoma induced in mice. Although iPSCs improved salivary gland function detected by a significant increase in the gene expression of *α*-amylase, the glands retained some malignant architecture including minor acinar, ductal, and vascular degenerative changes [123].

In an attempt to uncover the paracrine role of iPSCs in salivary gland regeneration, embryonic submandibular gland (SG) cells and mouse green fluorescent protein iPSCs (iSG) were cocultured. More developed epithelial structures were evident upon coculturing than in monoculture of embryonic SG cells. Upon morphological analysis of the regenerated tissues, iSG had a greater number of small acinar-like structures than that in SG cells. Additionally, analysis of differentiation markers among groups showed lower Sox2, c-Myc, and Nanog gene expression and higher Klf4 and Aqp5 gene expression in iSG with a remarkable regenerative capacity [124].

### 4.3. iPSCs and Periodontal Tissue Regeneration

iPSC differentiation into periodontal regenerative cells is affected by a variety of factors including cell source [125], culturing media [126], coculturing with inducing factors such as enamel matrix derivative (EMD) [127, 128], recombinant growth/differentiation factor-5 (GDF-5) [128, 129] or BMP-6 [130], the number of cellular passages [131], and type of scaffold used [130]. EBs generated from human gingival fibroblast and human neonatal skin fibroblast-derived iPSCs were induced into periodontal progenitor cells, which were then implanted on hydrogel scaffold subcutaneously in SCID rats. Owing to the cells' inherent epigenetic memory, iPSCs derived from gingival fibroblasts showed a higher expression of periodontal cell markers in vitro, including BSP, cementum protein 1 and periostin, and a formation of mineralized structure in vivo, with no teratoma formation observed with either cell types [125]. Neural crest cells derived from human skin fibroblast iPSCs cultured in combination with PDL cells' extracellular matrix showed a higher proliferation rate and a stronger expression of periodontal cell markers, including COL1A1, fibrillin-1, OPG, and periostin, as compared to cells cultured with either fibronectin, laminin, or dermal fibroblast extracellular matrix [126].

Culturing EB derived from human foreskin iPSCs in combination with EMD gel promoted the expression of RUNX2, an early osteogenic marker, but inhibited the expression of OCN, a late osteogenic marker, and mineralization in vitro. To assess the effect of iPSCs and EMD on osteogenic differentiation and periodontal regeneration in vivo, EBs derived from mouse iPSCs were seeded on apatite-coated silk fibroin scaffolds with EMD before implantation in the periodontal fenestration defect rat model. Following iPSCs-EMD in vivo transplantation, OCN, RUNX2, and OSX expression was higher than those in the control group which was attributed to the ability of EMD to recruit a large number of osteogenic cells. Moreover, iPSCs-EMD were able to induce the formation of new bone almost filling the periodontal defect, promoted the formation of new cementum covering the surface of the root, and stimulated the formation of periodontal fibers perpendicular to the root surface proving that iPSCs-EMD can further be an efficient tool in periodontal regeneration [127].

The periodontal differentiation potential of iPSCs derived from human gingival fibroblasts and treated with growth differentiation factor- (GDF-) 5 was investigated at different passages [5, 10, 15, 20]. All iPSCs-GDF-5-treated passages revealed a high proliferative ability and attained fibroblast-like cell morphology, significant production of calcified nodules, and upregulated expression of bone-related gene (OCN and BSP), periodontal ligament-related gene (periostin and vimentin), and cementum-related genes (cementum attachment protein and cementum protein 1) as compared to their untreated controls [131]. Yet, the periodontal differentiation capability of iPSC-derived MSCs, obtained either from human gingival tissues or from peripheral blood mononuclear cells, was significantly increased after their treatment with recombinant human GDF-5 (rhGDF-5) [128, 129]. This was confirmed by the marked expression of periodontal tissue-related genes (OCN, periostin, and cementum attachment protein). On the contrary, BM-MSCs treated with rhGDF-5 demonstrated an insignificant expression of periostin and CAP, despite the high expression of OCN. Similar results were attained upon loading PKH67-labeled iPSCs-MSCs-rhGDF-5 on hyaluronic acid and subsequent implantation into the dorsal surface of 6-8-week-old male athymic nude mice. Moreover, after 4 weeks of culture with rhGDF-5, both BMSCs and iPSCs-MSCs showed noticeable mineralization with nodule formation [129]. Chitosan/gelatin/glycerol phosphate hydrogel 3D scaffold seeded with osteogenic-induced rat fibroblast-derived iPSCs and BMP-6 applied to periodontal defect created on the root surface of the maxillary first molar in rats significantly downregulated inflammatory cytokines interleukin 8 (IL-8), tumor necrosis factor alpha (TNF-*α*), and IL-1*β* and promoted bone and periodontal tissue regeneration [130]. Additionally, human foreskin iPSC-derived MSCs, clotted with fibrinogen and thrombin implanted in periodontal fenestration defect in SCID rats, also revealed a significant increase in newly formed mineralized tissue area percentage [132].

Mesenchymal stromal cells derived from tail-tip fibroblast iPSCs (iPSCs-MCs) revealed immunomodulatory capabilities of the periodontal inflammatory destruction, which may offer a potential therapeutic modality for periodontal disease. In this context, a bacterial-induced periodontitis mouse model was established through local application of *Porphyromonas gingivalis* into the oral cavity and its systemic administration, while an acute inflammation model was created via subcutaneous implantation of heat-killed *Porphyromonas gingivalis*-impregnated sponge in rats. Rats were treated by systemic injection of iPSCs-MCs into the tail vein seven days following periodontitis establishment or by local iPSCs-MCs administration into the implantation site. iPSCs-MCs showed a significant reduction in inflammation and alveolar bone loss in the periodontitis rats' model. Moreover, local or systemic iPSC treatment in the acute inflammation model showed a reduced expression of the proinflammatory cytokine CXCL1, while local iPSCs-MCs administration resulted in a significant reduction in the inflammatory score [133]. Similarly, periodontitis was induced around the maxillary first molar bilaterally in female rats by ligature and subsequent infection with *Porphyromonas gingivalis*. The rats were treated intravenously and topically with rat iPSCs-MSCs reprogrammed from rat embryonic fibroblasts and transduced with tumor necrosis factor alpha-stimulated gene-6 (TSG-6) (iPSCs-MSCs/TSG-6). A significant downregulated level of alveolar bone loss, a few number of TRAP-positive osteoclasts, and serum interleukin 1*β* (IL-1*β*) and tumor necrosis factor alpha (TNF-*α*) were demonstrated as compared to untreated rats [134].

### 4.4. iPSCs and Enamel Regeneration

Ameloblasts are crucial cell populations required for enamel formation. The ability of mouse iPSCs (miPSCs) to differentiate into ameloblast was investigated [135], where miPSCs cocultured with dental epithelial cells differentiated into ameloblasts, exhibiting epithelial cell-like morphology in addition to expressing ameloblastic markers (ameloblastin and enamelin) and epithelial markers (p63 and cytokeratin- (CK-)14), suggesting an epithelial-mesenchymal interaction role in tooth development. Similarly, miPSCs differentiated into ameloblast-like cells under feeder-free conditions, using cultured epithelial rests of Malassez (ERM) cell CM and gelatin-coated dishes [136]. The differentiated ameloblast-like cells demonstrated an increase in expression of CK-14, amelogenin, and ameloblastin in comparison to miPSCs cocultured with ERM cells. The levels of amelogenin expression in ameloblast-like cells were significantly higher than those in miPSCs cocultured with ERM cells throughout the experiment, while ameloblastin increased significantly on day 14. Moreover, the addition of neurotrophin-4 to miPSCs under serum-free culture conditions during EB formation leads to their differentiation into dental epithelial-like cells with the upregulation of epithelial and ameloblastic markers [137]. These studies highlighted the potential differentiation ability of iPSCs into ameloblasts confirming that iPSCs could be a new cell source for enamel regeneration.

### 4.5. iPSCs and Dentin Pulp Complex Regeneration

The generation of odontoblast cells from iPSCs could open new opportunities for treating dentinal and/or pulpal damage. Epithelial-mesenchymal interactions are required for differentiating iPSCs into odontoblasts. Herein, the study described the “hanging drop” technique for differentiating miPSCs into odontoblast-like cells exploiting such an interaction. iPSCs were differentiated into EBs and then cultured on a collagen scaffold (CS) in combination with BMP-4 (CS/BMP-4). The generated cells intensely expressed mature odontoblast markers, dentin sialoprotein (DSP), and dentin matrix protein-1 (DMP-1) and presented physiological as well as functional features of odontoblasts [138]. Moreover, in an in vitro model, matrix metalloproteinase- (MMP-) 3 small interfering RNA was transfected into odontoblast-like cells derived from iPSCs. Strikingly, treatment with inorganic polyphosphate induced MMP-3 that physiologically accelerated both proliferation and differentiation of odontoblast-like cells, thereby hypothesized to provide some protection to the cells against the detrimental effects of inflammation and pulp capping materials. Additionally, DSPP and DMP-1 mRNA expressions were upregulated [139].

Under modified culture protocols, miPSCs were differentiated into neural crest-like cells (NCLCs) that could further differentiate into iPSC-derived dental mesenchymal cells (DMC) including odontoblast progenitor cells. Results showed that iPSC-derived NCLC expressed NC markers as demonstrated by immunocytochemistry, flow cytometry, and RT-PCR. Furthermore, NCLC expressed MSC markers, in addition to Pax9 and DSP, proving their capacity to differentiate into dental mesenchyme, when cultured with dental epithelium [140]. Interestingly, gene transfection of Pax9 and BMP-4 into iPSC-derived NCLCs promoted their differentiation into odontoblast-like cells, thus prompting signaling modulation of DMP-1 and DSPP expression, associated with odontoblastic differentiation of miPSCs [141]. In another study, dental pulp stem cells (DPSCs) were reprogrammed into iPSCs; then, the cells were seeded on dentin discs with PLLA scaffolds and implanted subcutaneously in mice. Amazingly, iPSCs generated a pulp-like tissue having tubular dentin, while in vitro, iPSCs maintained the odontogenic and mineralization potential after long-term expansion opposite to DPSCs [142].

### 4.6. iPSCs and Whole Tooth Regeneration

In addition to ameloblastic and odontoblastic differentiation potential of iPSCs, the capability of iPSCs in whole tooth regeneration was investigated [143–145]. miPSCs which clearly express odontogenic and osteogenic genes following their induction were implanted combined with epithelial and mesenchymal cells in a tooth germ model and transplanted into subrenal mouse capsule [145]. After four weeks of implantation, the formation of bone, dental pulp-like, and irregular tooth-like structures was demonstrated. Additionally, OPN was expressed in the apical region of the tooth-like structure. Notably, implantation of miPSCs alone failed to form dental or bone-like structures in contrast to its combined implantation with epithelial and mesenchymal cells.

Human iPSCs, derived from urine cells, were differentiated into epithelial sheets and cocultured with mouse dental mesenchyme, demonstrating an ability to form tooth-like structures such as enamel organ, enamel space, dentin, and dental pulp with physical and chemical properties similar to human teeth [143]. Further, through specific human antigen expression, it was revealed that iPSC epithelial sheets differentiated into ameloblast, while dental mesenchymal cells gave rise to the rest of the formed dental tissues. Interestingly, mouse dental mesenchymal cells alone formed bone-like tissue rather than tooth-like structure. Furthermore, miPSCs cultured in ameloblast serum-free CM supplemented with BMP-4 displayed the ability to form ameloblast- and odontoblast-like cells [144]. In addition, ameloblast serum-free CM increased the gene and protein expression of enamelin, ameloblastin, and CK-14, as well as phosphorylated Smad1/5, p38 MAPK, and ERK1/2 MAPK in miPSCs as compared with miPSCs cultured in epithelial cell medium for 14 days. Smad1/5 signaling transduction regulates the ameloblastic differentiation of miPSCs induced by ameloblast serum-free CM as the inhibition of Smad1/5 phosphorylation significantly reversed the increased the previously mentioned expression profile [146]. These results raise the possibility of iPSCs' use in whole tooth engineering opening a new gateway for biological tooth replacement.

## 5. Challenges Facing iPSCs' Human Clinical Applications

One of the major drawbacks that could hinder iPSCs' clinical application is their reported chromosomal instability and the underlying risk of tumor formation, which constitutes a substantial health hazard [12, 147]. Undifferentiated iPSCs' pluripotency and their ability to differentiate into tissues derived from the three germ layers are an incentive to teratoma formation, which is used as an assay to test their pluripotency [33, 148]. Moreover, iPSCs express several oncogenic genes [149]. Owing to iPSCs' unique properties, the generated tumor properties and origin are highly unpredictable and vary with the transplanted cell number as well as the utilized cell line [150]. In addition to their innate tendency for teratoma formation, the method of gene transduction can also increase the risk of tumorigenesis particularly due to the use of viruses that integrate their genome into the reprogrammed cells, as previously discussed. Currently, several attempts are carried out to overcome this through the use of nonviral vectors [25, 26] but are hindered by their lower transfection efficacy, especially following iPSC passaging.

Luckily, utilizing terminally differentiated iPSCs prior to implantation in addition to using nonviral vectors can help reduce risk of tumor formation [151]. Moreover, iPSCs can be reprogrammed via Oct3/4, Sox2, and Klf4, while omitting c-Myc which is a potent oncogene [14, 15, 152]. However, even following iPSC terminal differentiation, some cells may escape differentiation. Residual undifferentiated or partially differentiated iPSCs in the cellular transplants may cause teratoma formation upon implantation in the recipient tissues [153, 154]. Furthermore, iPSCs could retain epigenetic memory, which may affect their subsequent differentiation and direct them into lineages related to their parent cells [155, 156].

Another limitation associated with most current stem/progenitor cell isolation and expansion protocols lies in the utilization of xenogeneic-derived products in iPSC protocols. iPSCs are usually cultured on xenogeneic feeder cells that maintain the cells in an undifferentiated state without affecting their pluripotency [157], as well as fetal bovine serum that represents an important culture medium constituent [151, 158]. Using xenogeneic products in clinical trials could elicit an immunogenic reaction, carry a risk of disease transmission [151, 158], and affect reproducibility, as the exact composition of bovine serum varies greatly [159]. An additional problem creating an obstacle for the clinical application of iPSCs is the reduced generation efficacy [23], where iPSC generation efficiency using fibroblasts is extremely low. Even though generation efficiency is 4 to 10 times greater using dental pulp stem cells than fibroblasts, it is still relatively low for application in regenerative medicine [151].

## 6. Short- and Long-Term Perspectives of iPSC-Mediated Tissue Regeneration

Despite that iPSCs have shown promising results in regenerative medicine, a number of issues are yet to be resolved to allow their translation into clinical application while minimizing their potential side effects. Coculturing iPSCs with cells and growth factors could provide a promising solution to overcome tissue engineering challenges through mimicking in vivo conditions to optimize tissue regeneration results. Upon coculturing iPSCs-MSCs with iPSCs-macrophages committed to osteoblastogenesis and osteoclastogenesis, an OPG/RANKL milieu could be provided [80]. Similarly, coculturing iPSCs with dental epithelial and mesenchymal cells can reproduce epithelial-mesenchymal interaction signals orchestrating the process of tooth development. So far, securing an epithelial-mesenchymal interaction represents a great obstacle in whole tooth regeneration [135, 143]. Epithelial-mesenchymal interaction signals thereby remain to be the key towards inducing the differentiation of iPSCs into ameloblasts and other dental cells, which is the first step in whole tooth regeneration. Moreover, defining the best combination of iPSCs, signaling molecules such as growth factors, and scaffold biomaterials and determining the ideal architectural design of the scaffold 2D or 3D, sphere- or rod-shaped, remain crucial for various applications of iPSCs in dental and paradental tissue regeneration.

Transduction of repaired, edited, and/or modified genes in iPSCs could be a beneficial tool for treating various disorders. In this context, repairing RUNX2 gene mutation in iPSCs derived from cleidocranial dysostosis patients [52] as well as transducing nuclear matrix protein SATB2 [104] and Alox5 gene into iPSCs promoted osteodifferentiation [115]. Besides, Pax9 and BMP-4 gene transfection into iPSC-derived NCLCs promoted odontoblast-like cell differentiation [141] and attained a long-term effect of these factors rather than the short-term effect acquired following their local application [113].

iPSCs' extracellular vesicles, containing protein, mRNA, and miRNA, can further be used in regenerative medicine, seizing the paracrine effect of iPSCs while avoiding the possible risk of tumorigenesis associated with iPSC-based therapy [160]. The paracrine role of iPSCs in salivary gland regeneration has been proven upon coculturing embryonic submandibular gland cells and mouse iPSCs [124]. Besides, human iPSC CM promoted the osteogenic differentiation of human MSCs [122]. Usage of iPSC-derived secretome for tissue regeneration merits further research such as determining the active genes and growth factors expressed in CM from iPSCs.

Defining the optimum and the most accessible cell source to attain iPSCs should be investigated in the future to maximize their differentiation potential as well as their generation efficacy. iPSCs proved to retain their epigenetic memory, which may affect their subsequent differentiation [155, 156]. For example, iPSCs derived from gingival fibroblasts showed a higher expression of periodontal cell markers in vitro [125]. This could be beneficial in using particular cell sources for specific tissue regeneration, but it hinders the wide range of cells that could be derived from iPSCs. Despite that gingival fibroblasts and urine cells could be considered an easy source for attaining iPSCs, the generation efficiency of iPSCs using fibroblasts is extremely low [151].

A better control of the differentiation potential of iPSCs could be achieved by defining the suspension time of EB, since iPSCs-MSCs obtained at early EB suspension time possessed a more stem cell phenotype while those cells obtained later acquired a more differentiated phenotype [68], and by controlling and optimizing the reprogramming method where the highest cell density was attained in Sendai-iPSCs, while retro-iPSCs showed poor osteogenic differentiation [86].

Finally, next-generation sequencing could be alternatively used to assess the pluripotency potential, following iPSC generation instead of the complicated current techniques including teratoma formation and in vitro embryoid body (EB) generation [32].

## 7. Conclusion

iPSCs represent an autologous cell source, derived from the patient's own tissue, with no risk of immune reaction [161–163]. They have higher proliferative rates than adult stem cells and can be acquired via noninvasive methods [161], all properties that are highly desirable in regenerative medicine. Despite challenges associated with iPSCs' clinical use, their potential impact on medical applications still warrants further research. Carrying the application of iPSCs for tissue regeneration into humans entails strict abiding to the conduct of good manufacturing practice (GMP), as well as properly selecting cell source, culturing media, and vectors for gene transduction and excluding any xenogeneic-derived products from iPSC generation protocols. Recently, iPSCs have been successfully generated using a protocol compliant with GMP from hematopoietic stem cells from peripheral blood [164]. Furthermore, iPSCs were successfully maintained undifferentiated in xenogeneic-free culture medium and were subsequently differentiated into MSCs and osteogenic cells. Positive results were also attained following implantation in rats' calvarial defects [165] which paves the way for carrying iPSCs into clinical trials. Initial reports documented that the risk of teratoma formation associated with iPSC transplantation could be inhibited by pretreatment with resveratrol [112] or by irradiation of 2 Gray (Gy) prior to transplantation [43]. Finally, iPSCs' extracellular vesicles and secretomes, containing protein, mRNA, and miRNA, can alternatively be used, exploiting the paracrine effect of iPSCs while avoiding the risk of tumorigenesis associated with iPSC-based therapy [160].

It can thus be concluded that even though iPSCs hold a tremendously unexplored potential in the field of regenerative medicine, bone and dental tissue engineering, therapeutic application in bone disorders, gene therapy, and personalized medicine, a number of obstacles must be alleviated to attain their clinical applications. iPSCs still warrant further research focusing on achieving a safe, efficient reprogramming and attaining significant expansion while evading postimplantation tumor risks. Unleashing the full capabilities of iPSCs holds a promise of offering remedies to several genetic disorders in addition to their potential application in bone and dental tissue regeneration.

## Figures and Tables

**Figure 2:**
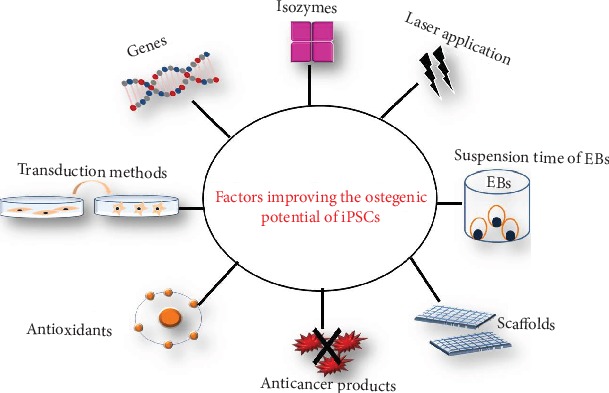
Diagram summarizing factors which may affect osteogenic potential of iPSCs.

**Table 1:** Studies investigating the regenerative potential of iPSCs.

Authors, year	Cell source	Study model	Scaffold	Outcome
Bone				
Tashiro et al., 2009 [24]	20D17, 38C2, and stm99-1 from mouse EF	In vitro In vivo	—	CA promoter potently transduced iPSCs with enhanced osteogenic differentiation.
Kao et al., 2010 [112]	Murine germ line-competent from rat EF	In vitro In vivo	—	Resveratrol had antiapoptotic effect and enhanced osteogenic differentiation of iPSCs.
Li et al., 2010 [60]	Mouse tail-tip fibroblasts	In vitro In vivo	—	Retinoic acid and TGF-*β* enhanced osteogenic differentiation of iPSCs.
Bilousova et al., 2011 [81]	Mouse dermal fibroblasts	In vitro In vivo	3D gelatin scaffold	3D gelatin scaffold enhanced functional osteoblastic differentiation of iPSCs.
Ye et al., 2011 [104]	Mouse tail-tip fibroblasts	In vitro In vivo	Silk scaffolds	SATB2 facilitated iPSC differentiation towards osteoblast lineage cells with enhanced bone formation and mineralization.
Hayashi et al., 2012 [43]	iPS-MEF-Ng-20D-17	In vitro In vivo	PES scaffolds	2 Gray irradiation prior to transplantation inhibited teratoma formation.
Levi et al., 2012 [100]	Human adipose-derived stromal cells	In vivo	HA-coated, BMP-2–releasingPLA scaffold	HA-coated, BMP-2–releasing PLA scaffold promoted osteogenesis.
Li and Niyibizi, 2012 [61]	Murine tail-tip fibroblasts	In vitro In vivo	HA/TCP scaffolds	TGF-*β* promoted iPSC-derived EBs towards osteogenic lineage.
Villa-Diaz et al., 2012 [165]	Human fibroblasts	In vitro In vivo	Poly [2-(methacryloyloxy) ethyl dimethyl-(3-sulfopropyl) ammonium hydroxide] scaffold	hiPSCs cultured in a xeno-free system can differentiate into MSCs and form bone in vivo.
Ardeshirylajimi et al., 2013 [53]	Human iPSC line	In vitro In vivo	PES scaffolds	Plasma-treated PES scaffolds promoted osteogenic differentiation of iPSCs.
Ardeshirylajimi et al., 2013 [48]	Human iPSC line	In vitro	PES scaffolds	PES scaffold enhanced differentiation of iPSCs into osteoblast-like cells.
de Peppo et al., 2013 [86]	11C and 1013A iPSC (dermal fibroblast), BC1-iPSC(bone marrow)	In vitro In vivo	Decellularized bone scaffold	Different reprogramming methods can influence osteogenic potential of iPSCs.
Jin et al., 2013 [106]	Cat SC101AiPSC	In vitro In vivo	Macrochanneled PCL scaffolds	iPSCs exhibited in vitro transcription and translation of osteogenesis-related molecules and in vivo bone induction.
Liu et al., 2013 [66]	Human B1 cell line	In vitro	CPC immobilized with RGD (Arg-Gly-Asp)	iPSCs transduced with BMP-2 showed enhanced osteogenic differentiation.
Nasu et al., 2013 [91]	Human BMSCs and DFs	In vitro	—	No difference was noticed in chondrogenic and osteogenic differentiation of iPSCs from different origins.
Thein Han et al., 2013 [72]	Human BC1 cell line	In vitro	Biofunctionalized CPC	Biofunctionalized CPC enhanced osteogenic differentiation and mineralization.
Zou et al., 2013 [90]	Human fibroblast	In vitro In vivo	PCL or PHT	Increased ALP activity and calcium deposition on PHT scaffold in vitro as well as ectopic bone formation in vivo in comparison to PCL
Ardeshirylajim et al., 2014 [97]	Human fibroblast iPSC lines	In vitro	—	iPSCs showed a higher capacity for osteogenic differentiation compared to AT-MSCs.
Dogaki et al., 2014 [93]	Mouse embryonic fibroblast	In vitro	—	iPSCs revealed higher osteogenic differentiation capability in comparison to BM-MSCs.
Hong et al., 2014 [87]	Rhesus macaques' BMSCs, skin fibroblasts, and CD34+ cells	In vitro In vivo	HA/TCP	iPSCs demonstrated robust bone formation.
Hynes et al., 2014 [88]	Gingival fibroblasts, periodontal ligament cells, and human lung	In vitro In vivo	HA/TCP	iPSCs derived from PDL showed a superior capability to form mature bone.
Lee et al., 2014 [58]	Human fibroblasts	In vitro	—	MSC CM enhanced osteogenic differentiation of iPSCs.
Liu et al., 2014 [67]	Human BC1 cell line	In vitro	CPC immobilized with RGD	NELL1 gene overexpression enhanced osteogenesis.
Kang et al., 2014 [89]	Human fibroblast	In vitro	PCL or PCL-nHA	Increased expression of osteogenic genes in both OC scaffolds was highly expressed in PCL-nHA in comparison to PCL scaffolds.
Kang et al., 2014 [107]	IMR90p18-iPS	In vitro	Mineralized gelatin methacrylate-basedmatrices	Osteogenic differentiation of hiPSCs was achieved through biomaterial-based cues alone.
Kanke et al., 2014 [114]	Human neonatal dermal fibroblasts Mouse fibroblasts	In vitro	—	An effective strategy for differentiation of mESCs, miPSCs, and hiPSCs into osteoblasts was deviced.
Ko et al., 2014 [57]	Human iPSC line (SC802A-1)	In vivo In vitro	HA/b-tricalcium phosphate scaffold Fibrin glue scaffold	iPSCs differentiated into functional osteoblasts and demonstrated bone regenerative effect comparable to human BM-MSCs in vivo. Osteoinduced hiPSCs showed relatively lower and delayed expressions of the osteogenic marker in vitro.
Ochiai-Shino et al., 2014 [109]	Human iPSCs (line 201B7) from adult fibroblasts	In vitro	—	TNAP-positive cell hiPSC-derived EBs expressed high levels of osteogenic genes.
Phillips et al., 2014 [99]	Human SFs (NIHi2 and NIHi7)	In vitro In vivo	HA/TCP	BM-MSCs cultured on HA/TCP promoted bone formation.
Tang et al., 2014 [77]	BC1 cell line	In vitro	CPC	CPC scaffold promoted osteoblastic differentiation.
Wu et al., 2014 [115]	Tail-tip mouse fibroblasts	In vitro In vivo	CCHS	Alox5 affects the osteogenic and adipogenic abilities of iPSCs.
Ardeshirylajimi and Soleimani, 2015 [110]	Human iPSC line	In vitro	—	Combination of OM and ELF-EMF promoted bone differentiation.
Ishiy et al., 2015 [94]	SHED and human dermal fibroblast	In vitro	—	Osteogenic potential of SHED-iPSCs and iPSCs-fibroblasts-iPSCsis higher than osteoinduced SHED.
Ji et al., 2015 [121]	Human gingival fibroblasts	In vitro In vivo	nHA/CG scaffolds	Sphere-nHA/CG increased hiPSC osteogenic differentiation and bone formation.
Kang et al., 2015 [95]	Human dermal fibroblast	In vitro	—	iPSCs showed osteogenic efficacy comparable to BM-MSCs.
Lepage et al., 2016 [96]	Equine fibroblast	In vitro	—	iPSCs showed early mineralization indicating early osteogenesis.
Wang et al., 2015 [73]	BC1 cell line	In vitro In vivo	RGD-coated macroporous CPC	Enhanced osteogenic differentiation of iPSCs
Wang et al., 2015 [105]	Umbilical cord mesenchymal cells	In vitro	Synthetic peptide-decorated 2D microenvironment via pDA chemistry and CMC	Peptide-decorated niche promoted osteogenic differentiation of human iPSCs.
Hayashi et al., 2016 [56]	Human iPSCs (line 201B7)	In vivo In vitro	Peptide nanofiber hydrogel scaffold	Increased bone regeneration using iPSCs delivered in the nanofiber scaffold.
Jeon et al., 2016 [80]	Dermal fibroblasts	In vitro In vivo	PLGA/PLLA	3D biomaterials promoted osteogenic differentiation of iPSCs.
Ji et al., 2016 [49]	Human gingival fibroblasts	In vitro In vivo	HCG	Osteogenic differentiation of hiPSCs was improved by HCG scaffold.
Kang et al., 2016 [102]	IMR90p18-iPS cell line	In vitro In vivo	Macroporous synthetic matrices	Adenosine induced hiPSC differentiation into functional osteoblasts.
Sheyn et al., 2016 [68]	Dermal fibroblasts	In vitro In vivo	—	Genetic modification of iPSCs-MSCs and the suspension time of EB can effectively influence bone regeneration.
Sladkova et al., 2016 [76]	1013A cell line obtained from dermal fibroblasts	In vitro	Macroporous CPC using PEG particle	Enhanced osteogenic differentiation
Wang et al., 2016 [78]	Human BC1 cell line	In vitro	Injectable CPC with hydrogel fibers	Injectable CPC with hydrogel fibers promoted osteogenesis.
Wang et al., 2016 [79]	BC1 cell line and clone 1 from human foreskin fibroblast	In vitro	Injectable CPC with hydrogel fibers	Injectable CPC with cell-encapsulating hydrogel fibers was associated with enhanced bone regeneration.
Wang et al., 2016 [69]	Human BC1 cell line	In vitro In vivo	CPC alginate microbeads	Osteoinduction or transduction with BMP-2 promoted osteogenic differentiation.
Xie et al., 2016 [74]	Mouse MiPS-01 cell line	In vitro In vivo	Biomimetic nanofiber HA/Col/CTS	Biomimetic nanofiber HA/Col/CTS was associated with upregulation of osteogenic genes.
Zhang et al., 2016 [85]	Human foreskin fibroblasts	In vitro In vivo	Porous *β*-TCF	Dimethyloxaloylglycine promoted iPSC angiogenesis.
Chijimatsu et al., 2017 [92]	Mouse neural crest cells	In vitro In vivo	—	iPSCs failed to repair rat osteochondral knee defects although chondrogenic and osteogenic capacity in vitro was comparable to human BM-MSCs.
Deng et al., 2017 [101]	hNF-C1 line obtained from dermal fibroblasts	In vitro	Peptide-conjugated nanofiber scaffold	Nanofiber scaffolds facilitated osteodifferentiation of hiPSCs.
Liu et al., 2017 [64]	Human BC1 cell line	In vitro In vivo	CPC	HUVECs promoted mineralization of iPSCs.
Ma et al., 2017 [51]	E14 mouse embryonic fibroblasts	In vitro	—	ES and iPSCs were similar in their osteogenic differentiation potential.
Zhang et al., 2017 [65]	Human BC1 cell line	In vitro In vivo	CPC	HUVECs and pericytes promoted mineralization of iPSCs.
Chen et al., 2018 [63]	Human BC1 cell line	In vitro In vivo	CPC	HUVECs promoted mineralization of iPSCs.
Oudina et al., 2018 [122]	Human adult myoblasts	In vitro In vivo	Coral scaffold	Undifferentiated hiPSC implantation promoted the formation of bone-like structures of murine origin.
Saito et al., 2018 [52]	Oral mucosa of 2 CCD patients	In vitro In vivo	Peptide nanofiber scaffold	Repairing RUNX2 mutation in iPSCs-CCD promoted osteogenesis.
Wang et al., 2018 [62]	Human BC1 cell line	In vitro	CPC	Metformin promoted osteogenic differentiation of iPSCs.
Wu et al., 2018 [70]	Human foreskin fibroblasts	In vitro In vivo	Injectable alginate microbeads	3G7 promoted antibody-mediated osseous regeneration.
Abazari et al., 2019 [120]	Human iPSC line	In vitro	PVDF/Col/PRP scaffolds	PRP-incorporated PVDF/col promoted iPSC osteogenesis.
Abazari et al., 2019 [47]	Human iPSC line	In vitro	PCL-PVDF (bFGF)	Incorporating bFGF in PCL-PVDF scaffolds promoted osteogenesis.
Al-Wahabi et al., 2019 [75]	Mouse MEF-NG-20D-17 cell line	In vitro	Polystyrene substrate	Different scaffold topography enhanced osteogenic differentiation.
Hosseini et al., 2019 [117]	Human iPSC line from HEK293T cells	In vitro	PHBV nanofiber scaffold	Nanofiber-based PHBV increased osteogenic differentiation.
Hosseini et al., 2019 [118]	Human iPSC line from HEK293T cells	In vitro	PCL-PLLA (poly-P) electrospun scaffolds	Poly-P in PCL-PLLA enhanced osteogenesis.
Kawai et al., 2019 [103]	414C2 and 409B2: human fibroblasts 1231A3: human PBMC 317-12: human fibroblast OI#1: skin fibroblasts OI#2: skin fibroblasts	In vitro In vivo	—	Retinoic acid induced the osteogenic differentiation iPSCs and bone formation.
Mao et al., 2019 [98]	Adipose-derived stem cells	In vivo	nHP gelatin cryogel scaffolds	ASC-iPSCs showed osteogenic differentiation.
Mirzaei et al., 2019 [116]	Human iPSC line from HEK293T cells	In vitro	2D and 3D PVDF	3D scaffold enhanced differentiation of bone-forming cells.
Ramaraju and Kohn, 2019 [71]	Human fibroblasts	In vitro In vivo	Mineralized scaffolds coated with DPI-VTK	Enhanced osteogenesis and angiogenesis
Saburi et al., 2019 [119]	Human iPSC line from HEK293T cells	In vitro	GO-PVDF	GO significantly improved osteoconductivity of the PVDF.
Sladkova et al., 2019 [108]	Mesenchymal progenitors derived from pluripotent stem cell line 1013A (1013A-MPs)	In vitro	Decellularized cow and human bone scaffolds	Both scaffolds equally supported cell viability, tissue growth, and formation of mineralized bone matrix.
Tahmasebi et al., 2019 [50]	Human iPS cell line	In vitro	PCL nanofibers with miRNA-22 and miRNA-126	miRNAs incorporated in PCL scaffold promoted osteogenesis.
Xu et al., 2019 [55]	Human fibroblasts	In vitro In vivo	HA derived from PLCL with peptide H1 in a core silk fibroin	Increased proliferation and osteogenic differentiation of iPSCs as well as fast bone formation in vivo
Zhong et al., 2019 [59]	Murine iPSCs derived from MiPS-01	In vitro	—	Osteoblast conditioned medium enhanced osteogenic differentiation.
Zhu et al., 2019 [54]	Human embryonic kidney line 293T	In vitro In vivo	—	Gene profiles of ESC and iPSC-derived osteoblasts are similar.
Salivary glands				
Alaa El-Din et al., 2019 [123]	Human skin fibroblasts	In vitro In vivo	—	iPSCs treated salivary gland carcinomas.
Ono et al., 2015 [124]	Stomach cells	In vitro In vivo	—	iPSCs accelerated salivary gland development and regeneration.
Periodontal tissues				
Duan et al., 2011 [127]	iPSCs (foreskin)-1-DL-1 from human foreskin fibroblasts	In vitro In vivo	Silk scaffold	EMD combined with iPSCs enhanced periodontal tissue regeneration.
Hynes et al., 2013 [132]	Human foreskin	In vitro In vivo	Fibrinogen and thrombin	iPSCs-MSCs enhanced periodontal tissue regeneration.
Yang et al., 2014 [134]	Rat embryonic fibroblasts	In vitro In vivo	—	iPSCs transduced with TSG-6 were associated with decreased inflammation and alveolar bone loss.
Yin et al., 2016 [128]	Human gingival fibroblasts	In vitro In vivo	—	EMD and GDF-5 induced periodontal differentiation of iPSCs.
Li et al., 2017 [131]	Human gingival fibroblasts	In vitro	—	Increasing culturing time had no effect on periodontal differentiation potential of iPSCs.
Yin et al., 2017 [129]	Peripheral blood mononuclear cells	In vitro In vivo	Hyaluronic acid hydrogels	rhGDF-5 promoted periodontal differentiation of iPSCs-MSCs.
Chien et al., 2018 [130]	Rat fibroblasts	In vitro In vivo	G/C/GP hydrogel phosphate	BMP-6-iPSCs on hydrogel scaffold promoted periodontal tissue regeneration.
Hamano et al., 2018 [126]	Skin fibroblasts	In vitro	—	iPSC-NCLC-PDL cells showed upregulated expression of periodontal tissue-related genes.
Hynes et al., 2018 [133]	Tail-tip fibroblasts from NOD/Lt mice	In vitro In vivo	—	iPSCs decreased inflammation and periodontal tissue destruction.
Li et al., 2018 [125]	Human gingival fibroblasts and human neonatal skin fibroblast	In vitro In vivo	Hydrogel	Gingival iPSCs demonstrated better expression of periodontal cells' markers.
Enamel				
Arakaki et al., 2012 [135]	Mouse embryonic fibroblast	In vitro	—	iPSCs cocultured with dental epithelial cells differentiated into ameloblasts.
Yoshida et al., 2015 [136]	Mouse embryonic fibroblast	In vitro	—	iPSCs differentiated into ameloblast-like cells cultured with epithelial cell rests of Malassez cell conditioned medium and gelatin-coated dishes.
Abdullah et al., 2019 [137]	Mouse embryonic fibroblast	In vitro	—	Neurotrophin-4 in addition to iPSCs promoted its differentiation into dental epithelial-like cells.
Dentin pulp complex				
Otsu et al., 2012 [140]	Mouse embryonic fibroblast	In vitro	—	iPSCs differentiated into NCLC could further differentiate into iPSC-derived dental mesenchymal cells including odontoblasts.
Ozeki et al., 2013 [138]	Mouse embryonic fibroblast	In vitro	Collagen type I scaffold combined with BMP-4	iPSCs differentiated into functional odontoblast-like cells.
Ozeki et al., 2015 [139]	Mouse iPSC line (iPS-MEF-Ng-20D-17)	In vitro		Treatment with inorganic polyphosphate induced MMP-3 that physiologically accelerated both the proliferation and differentiation of odontoblast-like cells derived from iPSCs.
Seki et al., 2015 [141]	Mouse iPSCs	In vitro	—	Gene transfection of Pax9 and BMP-4 into iPSC-derived NCLCs promoted their differentiation into odontoblast-like cells.
Xie et al., 2018 [142]	Dental pulp stem cells	In vitro In vivo	Dentin discs with PLA scaffolds	iPSCs cultured on dentin discs with PLA scaffolds formed pulp-like tissue with the presence of tubular dentin.
Whole tooth regeneration				
Wen et al., 2012 [145]	Mouse embryonic fibroblast	In vitro In vivo	Collagen hemisphere	iPSCs combined with epithelial and mesenchymal cells formed bone and dental pulp-like structures.
Cai et al., 2013 [143]	Human urine cells	In vitro In vivo	—	iPSCs cocultured with mouse dental mesenchyme formed tooth-like structure.
Liu et al., 2016 [144]	Mouse iPSC line (C5 cell line)	In vitro In vivo	Fibrin gel	iPSCs cultured in ameloblast serum-free conditioned medium supplemented with BMP-4 differentiated into ameloblast- and odontoblast-like cells.
Liu et al., 2020 [146]	Mouse iPSC line (C5 cell line)	In vitro	—	Ameloblasts serum-free CM increased the gene and protein expression of enamelin, ameloblastin, and CK-14, as well as phosphorylated Smad1/5, p38 MAPK, and ERK1/2 MAPK in miPSCs as compared with miPSCs cultured in epithelial cell medium for 14 days.

## Abbreviations

ALP: Alkaline phosphatase
ASC-iPSCs: Adipose-derived induced pluripotent stem cells
AT-MSCs: Human adipose tissue
bFGF: Basic fibroblast growth factor
BM-MSCs: Bone marrow mesenchymal stem cells
BMP: Bone morphogenetic protein
BMSC: Bone marrow stromal cells
CA: Cytomegalovirus enhancer/b-actin
CCD: Cleidocranial dysostosis
CCHS: Collagen/chitosan/hydroxyapatite scaffolds
CM: Conditioned media
CMC: Carboxymethyl chitosan
Col2.3GFP: 2.3 kb type I collagen promoter-driven green fluorescent protein
CPC: Calcium phosphate cement
DFs: Dermal fibroblasts
DPI-VTK: Dpiyalswsgma-Vtkhlnqisqsy
EBs: Embryoid bodies
EF: Embryonic fibroblasts
ELF-EMF: Extremely low-frequency electromagnetic field
EMD: Enamel matrix derivatives
ES: Embryonic stem
G/C/GP: Chitosan/gelatin/glycerol phosphate
GDF-5: Growth/differentiation factor-5
GO: Graphene oxide
HA/Col/CTS: Hydroxyapatite/collagen/chitosan
HA/TCP: Hydroxyapatite/tricalcium phosphate
HA: Hydroxyapatite
HCG: Nanohydroxyapatite/chitosan/gelatin
HEK: Human embryonic kidney
hiPSCs: Human induced pluripotent stem cells
HUVECs: Human umbilical vein endothelial cells
iPSCs: Induced pluripotent stem cells
iPS-NC-PDL cells: iPSCs induced into neural crest- (NC-) like cells
iPS-NC cells: p75 neurotrophic receptor-positive cells were cultured on extracellular matrix (ECM) produced by human PDL
MSCs: Mesenchymal stem cells
NCLCs: Neural crest-like cells
nHA/CG scaffolds: Nanohydroxyapatite/chitosan/gelatin scaffolds
nHA: Nanohydroxyapatite
OM: Osteogenic media
PCL: Polycaprolactone
PCL-PLLA: Polycaprolactone-poly-L-lactic acid
PCL-PVDF: Polycaprolactone-polyvinylidene fluoride
pDA: Polydopamine
PBMC: Peripheral blood mononuclear cell
PDL: Periodontal ligaments
PEG: Polyethylene glycol
PES: Polyethersulfone
PHT: Polymer hyaluronan and ceramic tricalcium phosphate ceramic particles
PLA: Poly-L-lactic acid
PLCL: Poly (L-lactic acid-co-*ε*-caprolactone)
PLGA/PLLA: Poly lactic-co-glycolic acid/poly L-lactic acid
Poly-P: Polyphosphate
PVDF: Polyvinylidene fluoride
PVDF/Col/PRP: Polyvinylidene fluoride/collagen/platelet-rich plasma
RGD: Arg-Gly-Asp
RUNX2: Runt-related transcription factor 2
SHED: Human exfoliated deciduous teeth
TGF-*β*: Transforming growth factor-beta
TNAP: Tissue-nonspecific alkaline phosphatase
TSG-6: Tumor necrosis factor alpha-stimulated gene-6.
